# Reconstructing Fashion: The Mock-Velvet Doublet of a Seventeenth-Century Florentine Waterseller

**DOI:** 10.1080/00404969.2024.2373863

**Published:** 2025-01-16

**Authors:** Sophie Pitman

## Abstract

This article explores an extraordinary ‘doublet of black stamped mockado, nasty’ owned by an everyday artisan, the Florentine waterseller Francesco Ristori, who died in 1631. Our only record of this garment — like many non-elite objects — is a written description in a posthumous inventory, but this article shows how we can reconstruct this doublet through a combination of archival, visual, material sources and hands-on experimental methods. Offering a thorough account of the processes and methodological basis of the material reconstruction, it explains why Ristori’s doublet exemplifies many key features of early modern everyday fashion, the historical importance of the doublet as a garment, the innovation of mixed fibre fabrics like mockado, the novelty of stamping techniques, the challenges and importance of black dye, and what the ‘nasty’ condition suggests about Ristori’s use of the doublet. It will also suggest why a Florentine waterseller might want to look fashionable, and will give an overview of Ristori’s domestic and working circumstances, in order to connect his clothing choices to his everyday experiences. This micro-history of Ristori’s doublet brings to life the macroworld of early modern everyday fashion.

## Introduction

When the Florentine waterseller Francesco Ristori died in 1631, a pandemic year in Italy, his belongings were catalogued and recorded in an inventory. From four handwritten pages, which list the contents of his rented house in Santo Spirito and a villa near a fountain in San Miniato, we can build a picture of his material surroundings. Ristori’s wardrobe gives us a glimpse into the clothing and textile choices made by early modern artisans in one of the most fashion-conscious cities in Europe. Amongst his used shirts and felt hats, we discover that Ristori was wearing items made of novel textiles dyed in bright colours and decorated with trims that would have created a fashionable appearance suited to his status as an enterprising artisan. In his chests containing spun waste silk hose, a yellow woollen gown, and an outfit from Nîmes in France, we also discover that Ristori owned a doublet of stamped mockado that was in a nasty condition (*giubbone di mucaiardo nero cattivo stampato*).^1^

Few objects survive that represent the kinds of clothes owned by Ristori or his peers. Every single person in early modern Europe (excepting the very poorest) likely owned at least two sets of clothing at any given time in their life — but from the hundreds of thousands of garments that must have been made, just a few dozen survive.^2^ Textiles were worth so much, economically and practically, that they were cared for, mended, and re-shaped into new clothes. They could be pawned and sold second-hand, and then altered afresh into new garments. As they wore down and were no longer fit for wear, bright dyes could be extracted for repurposing as pigments, metal threads might be removed and melted down, and fabrics repurposed as stuffing, patching, household rags, and menstrual cloths. Even in tatters, linen fibres still held enough material value to be turned into paper.^3^ Those objects that do survive — most commonly saved because they were worn by an important or wealthy person — are usually compromised. Textiles often endure immense wear and tear, absorbing bodily fluids, and suffering damage from light, humidity, and insect infestation. Old clothes, stored in trunks in descendants’ attics or sold in antique shops, were adapted to fit the bodies and contemporary tastes of those invited to costume balls, particularly in the nineteenth century.^4^ Surviving garments are often altered, have poor provenance and are unrepresentatively elite.

This loss is not unique to the early modern period or to textiles. As Glenn Adamson has explained, ‘[o]ne of the key problems in the study of material culture is the phenomenon of loss. Indeed, when it comes to the material past, disappearance is the norm, and preservation is the exception’.^5^ This article explores how we might overcome this material loss, to show how reconstruction might help us fill gaps in the historical record and disrupt traditional narratives about the past. By making a doublet based upon an entry from the waterseller’s inventory, from fibre to fitting, we can restore the creativity, ingenuity, and material effect of textiles worn by early modern artisans.

## Reconstruction Methods and Early Modern Clothing and Textiles

For decades, pioneering makers have been studying extant textiles and clothing to understand cut, construction, materials, and skill, in some cases publishing patterns that enable readers with pattern-making skills to reconstruct historic garments.^6^ Such scholarship has been particularly important for re-enactors, museum staff who wish to display replicas, and the theatre and film industries. The methodology of reconstruction or ‘re-methods’ more recently has been expanded, embraced by a wide range of academic disciplines that use hands-on and digital methods to reconstruct historical objects in the service of larger research questions. This shift offers and prompts new models for recovering the innovations and material ingenuity of early modern dress and textiles through remaking.^7^ Interdisciplinary collaborative grant-funded projects on early modern making, such as The Making and Knowing Project (led by Pamela Smith at Columbia University) and the Artechne Project (led by Sven Dupré at Utrecht University), have paved the way for this work by showing how wide a variety of historical findings might come out of reconstruction projects.^8^ While neither of these projects focused on textiles, both have revealed much about how Renaissance textiles were made and used, particularly regarding black dye techniques, and the implications of this for art, cultural, social, economic, scientific, and political history.^9^ Other reconstructions of early modern clothes have revealed the physical impression and tactile effects of textiles and accessories. Ulinka Rublack and Jenny Tiramani have remade some of the elaborate garments — shirt, doublet, hose, and feathered headpiece — worn by the stylish Fugger accountant Matthaeus Schwarz, as depicted and described in his unique ‘Book of Fashion’. They show the visual and sensory impact of creative dressing, reinforcing the role of appearance as a political act in early modern Europe.^10^ Sarah Bendall has used surviving objects, visual sources, and textual records to reconstruct the structured undergarments worn by early modern women, revealing insights about physical presence and space as well as the construction of gender in the period.^11^ These reconstructions have largely focused on the textiles and clothing of elite early modern men and women, and have been informed by written instructions or descriptions of processes and materials, rich visual sources, and even surviving objects.

The Refashioning the Renaissance Project incorporated reconstructions and experimental hands-on methods in order to materialise the clothing and textile culture of the middling ‘artisanal’ classes and to explore how fashion was experienced by non-elites in early modern Europe. But evidence about the textiles and dress worn by the middling levels of society — by which we mean those skilled craftsmen and shopkeepers who were economically and socially positioned between workmen and day labourers on the one hand and merchants and lawyers on the other — is spread thinly across a wide range of sources and so there is no single informative source upon which to base a reconstruction.^12^ In other words, we could not start from a recipe or set of written instructions, a detailed visual source, or a surviving object as previous successful early modern reconstructions have done. This challenged us to put the methodology to the test — how far could we push reconstruction? What would happen when we tried to reconstruct objects that we know existed, but for which no one ‘ur’ object or source survives?

## Selecting the Object for Reconstruction

**Fig. 1.**
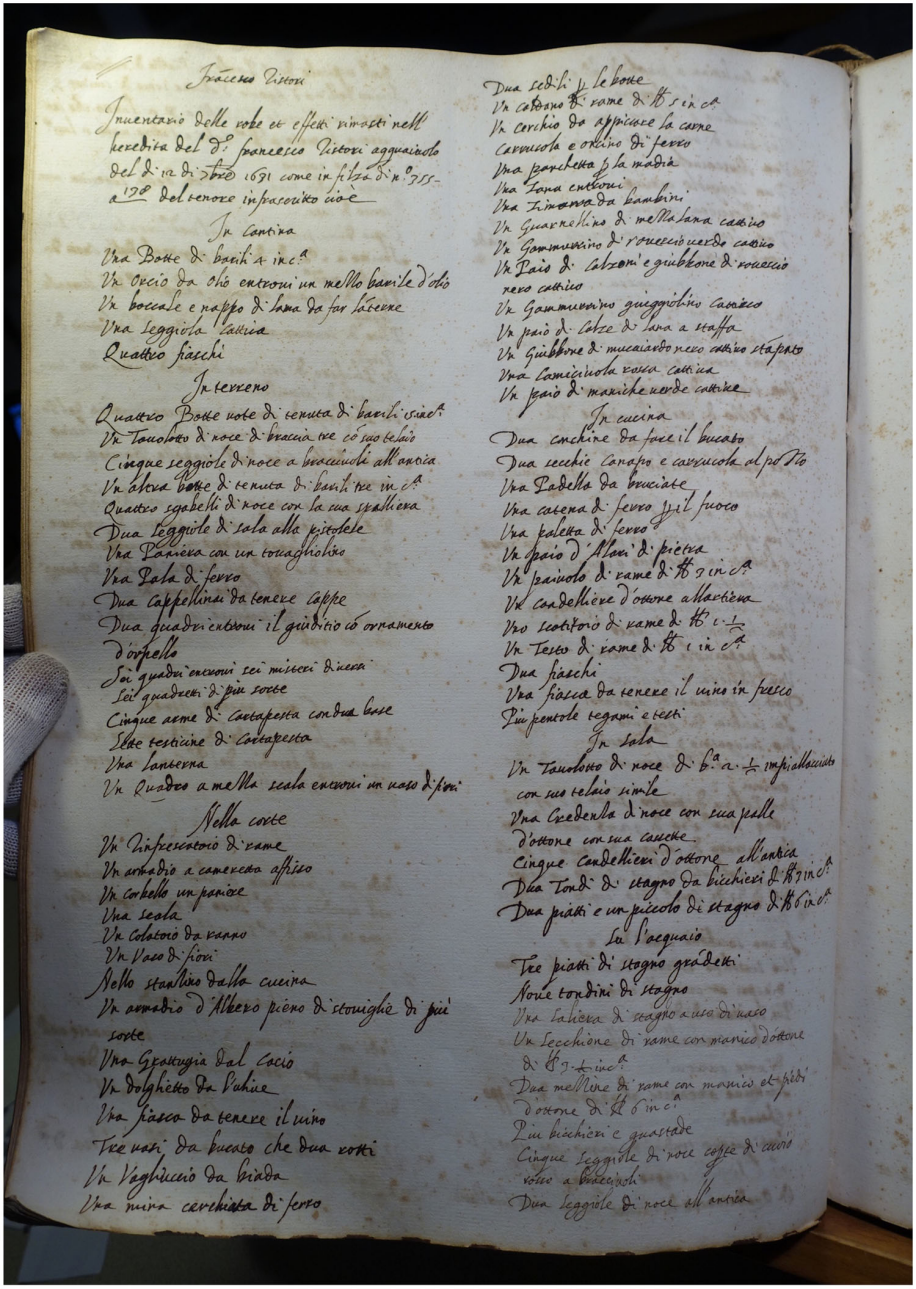
Inventory of Francesco Ristori, 1631, Magistrato dei pupilli, Archivio di Stato, Firenze, 2718/2, fol. 192v. The doublet is mentioned in the right-hand column, 14 lines down. Image taken by the Refashioning the Renaissance Project.

We turned to a set of 448 inventories, identified and transcribed by members of the Refashioning project, which record the possessions (including textiles, textile tools, and garments) owned by artisans in Florence, Siena, and Venice between 1550 and 1650. This data is now freely searchable in an open-access database of over 25,000 entries that record over 80,000 objects, assembled by the Refashioning project. The database offers an unprecedented overview of the textiles, hues, and decorations in garments worn by men, women, and children from the middling section of urban society, suggesting new insights into ownership patterns, fashion, and appearance.^13^ Could this data be used in order to fill in the material gaps in the historic record? By selecting just one entry, from one inventory, this reconstruction began with just seven words: — a doublet of black stamped mockado, nasty (*Un Giubbone d’ mucaiardo nero cattivo stampato*) (Fig. 1).

We selected this entry in part for its suggestive, if brief, description of an upper-body garment that seemed to represent novel, fashionable and, most crucially, middling forms of consumption from the era. Although this description seems comparatively sparse, on close reading it does tell us about the garment type, the fabric, the colour, the decoration, and even the state of repair, each of which were typical among our dataset, and either novel or undergoing a period of transformation during the early modern period (as will be further expanded below). We know the doublet was made of mockado fabric, it was black, it was decorated with a stamped pattern, and it was in a nasty condition. *Cattivo — *meaning ‘bad’ or ‘nasty’ — was a common qualitative statement in the inventory data, appearing eighty-four times.^14^ The term was used ambiguously in this entry, and so it might refer specifically to the quality of the dye or the cut of the doublet. But in the absence of other evidence, we interpreted it as a record of the overall condition of the doublet, as inventories were intended to assess economic value and general condition was frequently noted. A ‘nasty’ condition might suggest that the doublet had been worn regularly and had suffered from repeated use.

**Fig. 2.**
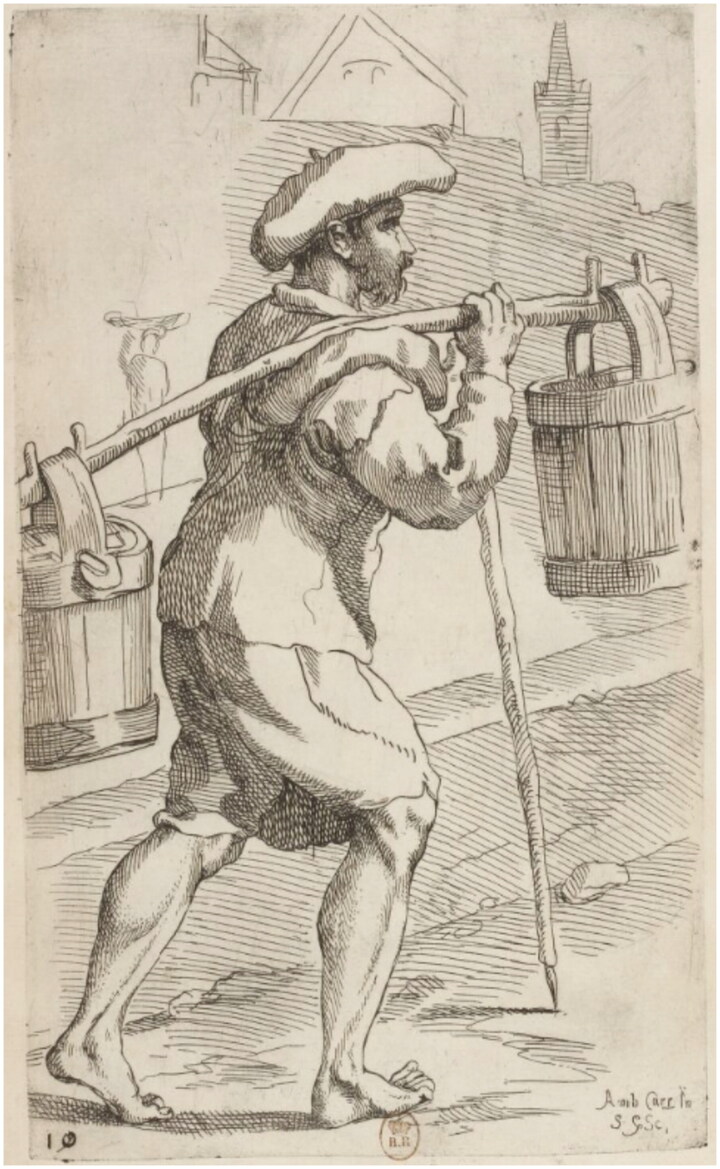
Simon Guillain after Annibale Carracci, *The Waterseller*, 1646, Bibliothèque nationale de France. Image in the public domain.

The owner of the doublet was a man called Francesco Ristori. The historical record reveals little about him (typical for the majority of Renaissance artisans who left few textual traces), except that when he died during a pandemic in 1631, he left behind a wife Maddalena, a *garzone* (apprentice) named Lorenzo, and a minor child or children, for his belongings were inventoried by the Office of Wards, responsible for protecting the interests of minors and widows.^15^ The court noted that he was a waterseller, and so Ristori likely earned his living by going house to house carrying fresh water, or selling it by the cup on the street. Watersellers were visible urban workers — we find them depicted in broadsheets of street cries and city scenes — so he was likely well known within the community (Fig. 2).^16^ He rented a villa with access to a fountain in San Miniato, and lived in the artisan district of Santo Spirito, located in the Oltrarno quarter on the south bank of the river Arno, a region largely occupied by artisans and workers. As Katherine Wentworth Rinne has argued, in Italy only the poorest families would carry their own water, and the richest families would have servants carrying it from private wells, so watersellers mostly catered to a middle market.^17^ Ristori likely served water to his peers, and so his doublet was probably seen and appreciated by those of a similar social and economic standing.

When writing about the structure of everyday early modern life, Fernand Braudel declared that ‘in every town in the world the water carrier was indispensable’.^18^ Cleanliness was a crucial element of sixteenth- and seventeenth-century healthcare, and so whether the water Ristori sold was for drinking, washing, laundry, or even the building trades, his clothing needed to signal that he was a respectable and trustworthy vendor with safe potable water.^19^ It seems unlikely that he wore this doublet at work, because it was among the finest of the 127 garments in the household at his time of death, and he would not have wanted it to get wet. Even if Ristori reserved this doublet for finest wear such as attending church or festive days, scholars of urban history have made clear just how much appearance was scrutinised and recalled within smaller networks of neighbours.^20^ Clothing was an important way of maintaining one’s personal and professional reputation. Ristori’s work probably provided him with the money to purchase the doublet, but the doublet also likely supported his occupational success.

Doublets, or in Italian *giubboni*, were upper-body garments worn by some women and most men across the social spectrum.^21^ Connected to a pair of hose or breeches with laces or hooks and eyes, and worn over a shirt, the doublet was an everyday item of clothing, part of a ‘suit’ of apparel. On the one hand, the doublet was a very ordinary Renaissance garment, but the English name referring to their double or multiple layers hints at the innovation of this item of clothing. Made by professional tailors, the doublet was often a garment in which craftspeople pushed their skills to the limit by using geometrically inspired pattern drafting and cutting techniques combined with sculptural inner linings to create dramatic and stylish silhouettes.^22^ Throughout the sixteenth and seventeenth centuries, doublets shifted in shape, style, and decoration. X-rays of surviving examples reveal the ways that innovative materials such as cork and stitching techniques were employed in creating doublets.^23^ Tailors also used pad stitching to shape shoulders and collars, and provided structure with quilted or stuffed wool, animal hair, bents or reeds, whalebone or baleen, card, and animal glues.^24^

The Refashioning database includes 1143 doublets across 771 descriptions, which record examples made of linen, wool, silk, mixed materials, and leather, in a wide range of colours (black, purple, yellow, red, white, brown, green, and grey) and with diverse decorative features (dotted, striped, slashed, flowered, embroidered, trimmed, and lined). While Ristori’s doublet was dyed black, the most popular colour recorded for Italian artisan doublets (201 examples), it was made of the comparatively unusual fabric mockado (7 examples). Only two other doublets are described as stamped. Therefore, this doublet was both popular and extraordinary, representative and yet unique. At once emblematic of everyday dress and yet made with novel Renaissance cut, materials, and methods, a reconstruction of a doublet could reveal a breadth of insights about the making and meaning of Renaissance everyday fashionable clothing.^25^

## Designing the Reconstruction: Skills, Expertise and Research Process

The doublet reconstruction project, which I led and coordinated in collaboration with the rest of the Refashioning project team and the School of Historical Dress, was designed with three strands: skill building, material reconstruction, and digital reconstruction. These processes were mutually informative: research findings in one strand often informed practice and development within another, but each used different methods and aimed for different results. The Refashioning team undertook skill-building training in order to get hands-on experience of each step in the making process that could inform our archival, visual, and material research. We learned to prepare and spin wool and linen at Leire in Sweden, tended silkworms and reeled silk from cocoons at Nido di Seta in Italy, tested dye recipes and materials at Columbia University in New York and Aalto University in Finland, learned to weave velvets at Lisio in Italy, and tailored half-size doublets and practised stamping with a hot tool at the School of Historical Dress in London. Given how little written evidence survives about how textiles and garments were made, it was essential that researchers gained hands-on experience in order to understand the tools, materials, and skills required at every step of making a doublet in as historically informed a way as possible — from fibre to finished garment. This helped us to appreciate the labour of textile workers, think critically about the ways that materials and techniques encourage or resist certain practices, and to make informed decisions about how to interpret our sources and translate that into the finished reconstruction. The goal of these experiments, workshops, and visits was to train us as researchers. As one of the core aims of the reconstruction was to demonstrate the fashionable achievements of the non-elites, who we could see from our data were often creatively dressing in materials, colours, and designs that suggested a distinctive urban middling culture that was distinct from but related to elite fashion, it was important that the fabric, dyes, decoration, and tailoring represent skilled work — and so we sought to commission and work alongside expert makers to create the full-size doublet.

But what does expertise mean in this context? Skilled twenty-first-century weavers, dyers, and tailors typically are not trained in historic methods of making, and so it is often necessary to accept compromise regarding site (whether the maker uses natural daylight and candles or artificial light, or electricity rather than a naked flame for heat, for example), material sourcing, and experience. While re-enactors and costumiers have often sought to represent textiles and dress worn by the labouring poor or ‘ordinary’ workers, few makers have had the opportunity or resources to work with middling fashion.^26^ Even the most proficient craftspeople are unlikely to have much experience working on clothing that would be suitable for a fashionable everyday middling artisan. In most cases, they make their living by creating costumes for theatre and film, or commissions for historic sites, and rely on published research that has tended to focus on the elites, so they have more knowledge and experience making objects fit for noblemen and women. Reconstruction projects also often must compromise on materials, using components created with industrial processes or synthetic materials, due to restraints on time, budget, and availability.^27^ Not only was it unusual for this project to be focusing on everyday fashions, but the focus on reconstructing a garment from raw materials with a generous budget and timeline required a novel approach and an open-minded and ambitious team.

It was clear that the doublet project had to be a collaborative dialogue between researchers and makers, so we turned to the School of Historical Dress, London (hereafter SHD) to make the doublet under the design and leadership of Jenny Tiramani.^28^ The opportunity to research this understudied section of society, as well as to work with hand-spun and handwoven fabrics, made this challenge an exciting opportunity for all the researchers and makers involved in the project. While the pandemic posed many logistic challenges, preventing us from working in proximity, slowing process and causing supply-chain issues, we all felt keenly that this was a unique opportunity to have significant time, budget, and creative freedom in collaborating on this doublet project, a rare privilege only won by support of major research grants. The third strand, digital reconstruction, is explored in a companion article in this journal issue.^29^

## Reconstructing Mockado Textile

The first step in the making process — spinning and weaving the fabric — was perhaps the greatest challenge both in terms of research and making practice. To begin, it was crucial to decipher the meaning of *mucaiardo* from Ristori’s inventory. Textile terminologies are notoriously challenging, and in the absence of a sample of the fabric attached to the inventory, we will never know exactly how the inventory scribe assessed each material.^30^ This launched a major research question: what was mockado, and how was it made? Guild records, sample books, and lexicons suggest that mockado was an imitation fabric, woven with supplementary warp loops in the same structure as silk velvet, but using a blend of wool, linen, hemp, and/or silk. The word is possibly derived from the Arabic *mukhayyar*, meaning ‘select, choice’, and possibly was etymologically confused with *mocajar*, a mid-quality camlet, and mohair. Mockado, and its many variant spellings and translations, were often elided with Naples fustian, tripp, and later moquette.^31^ Mockado was, therefore, a mock velvet (although this is a linguistic coincidence); much cheaper than silk velvet, but imitating the visual plush effect using less fine materials.

Ristori’s mockado represents one of the key product innovations in European textile manufacture, a shift towards lighter, colourful, and highly patterned textiles sometimes termed ‘new draperies’.^32^ These novel fabrics made of blends of different fibres in almost every imaginable combination and structure appealed to a wider market for economic and fashion reasons. Light and so cheaper to make and dye, they could be tailored into tight fits. Their makers, often inter-European immigrants fleeing religious persecution or seeking better markets for their products, could often cleverly skirt legal and guild regulations with novel combinations of fibres, and market them with fantastical and ever-changing names. In 1576, a London ordinance explained that because these new blended fabrics such as ‘tufted mockado’ were ‘not called by any of the same names that the cloths of woollen or linen of the former ancient time were (although they consist of the same substance)’, the makers and merchants were selling them without paying duties or following guild and city customs, a practice deemed ‘naughty and deceitful’.^33^ Likewise, buyers were more easily able to afford these fabrics, which often were permitted (or not yet included) in sumptuary laws, enabling them to achieve the fashionable looks and sensory effects formerly associated with the finest textiles restricted to elite wearers. In Pisa, for example, a 1563 law did not categorise mockado (*mocaiardo*) alongside wool (*panno*) or silk (*drappo*), even though it discussed the use of both wool and silk mockado fabrics (*mocaiardo di lana*, *mocaiardo di seta*). While men were forbidden from wearing mockado, artisan women were allowed one gown of silk mockado, even though they were forbidden from wearing *drappo* (silk).^34^ In Pistoia, 1562 regulations explicitly allowed mockado and camlet as alternatives to the banned *drappo*.^35^ In Mantua, mockado and *bavella* (fabric woven from threads made from spun silk waste) were allowed, although all other velvets were banned.^36^ Such laws reveal the conceptual confusion around mixed, imitative, and lower quality fabrics, but also how they were legally as well as culturally understood to be substitutes that could operate conceptually as well as sensorially to approximate luxury textiles.

Florentine sumptuary laws do not explicitly mention *mocaiardo*, suggesting that Ristori the waterseller and his peers could wear it freely. The textile is listed among goods that had to be taxed as they entered and exited the city in regulations drawn up in 1544, reformed in 1579 and augmented in 1652, attesting to the successful longevity of this textile. Double mockado from the Levant was taxed at six *lire* for between twenty and twenty-six *braccia*, while mockado coming from the West was only two *lire* for fifteen to sixteen *braccia*. Both fabrics were considered ‘a type of fabric of hair’.^37^ We found 294 objects made of mockado in our data from across the period — 238 of these were in Venetian inventories, only one from Siena, and fifty-five from Florence. Mockado was used in a wide range of objects from large items of apparel such as cloaks, gowns, petticoats, hose, and doublets to smaller pieces including sleeves and other accessories. These mockado garments are described as being worn by men, women, and children alike, and as having been dyed in many colours: most commonly black, but also brown, blue, green, red, white, pink, purple, and yellow. While previous narratives about Renaissance textiles typically separate wools, silks, and linens, the widespread use of these mixed blended fabrics suggests a more nuanced approach to textiles in the era is needed, particularly among the middling classes.^38^

**Fig. 3.**
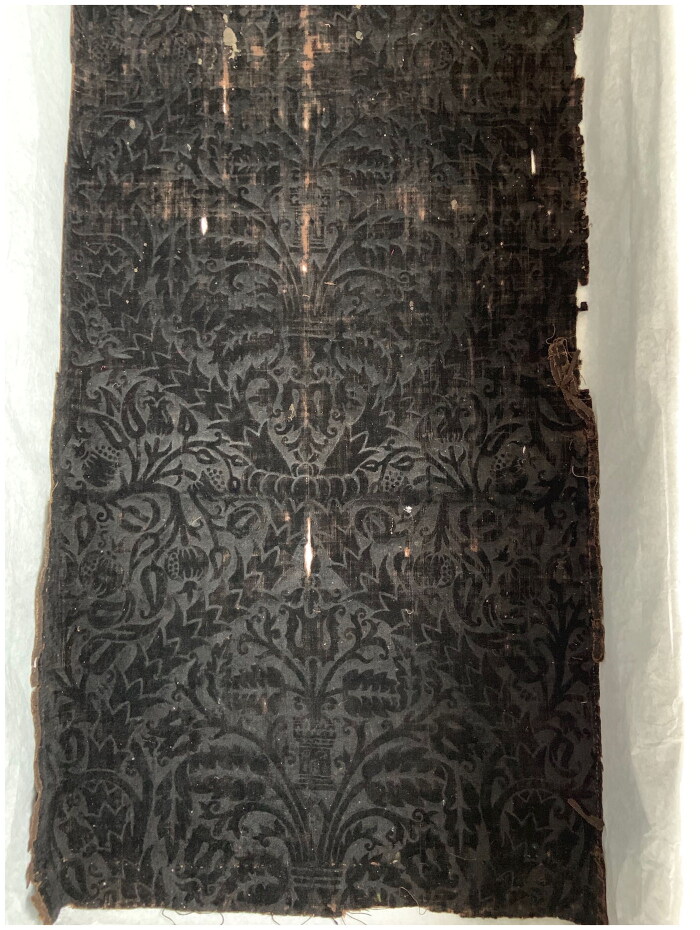
Detail of a piece of selvedge width wool velvet with a large stamped design. Photo taken by the School of Historical Dress Collection for the Refashioning the Renaissance Project.

Surviving examples of velvets made with wool, hemp, or linen, which might have been called mockado in the period, show just how effective these mixed textiles could be. Their dense plush pile and sheen is often so similar to pure silk velvet that it can be hard to identify their fibre composition without close analysis or scientific testing. In order to get specific information about thread count, fibre, and dyes, samples of the warp, weft, and pile warp of a stamped wool velvet were taken from a surviving example in the SHD collection, which was examined under microscope and using ultra-high performance liquid chromatography, high resolution mass spectrometry, and scanning electron microscopy by Cristina Carr at the Metropolitan Museum of Art, New York, Art Proaño Gabor at the Rijksmuseum, Amsterdam, and Krista Vajanto at Aalto University, Finland. From these results, we decided that our mockado would be made of a linen ground with supplementary wool pile, following the example from the SHD collection (Fig. 3).

Given the velvet weave structure of mockado, for which a metal rod is inserted by hand into alternate rows of weaving to create loops before the supplementary warp is cut with a razor, we first considered working with a silk velvet weaver. Velvet requires highly developed hand skills, and the work is risky and slow. However, because wool and linen have different material properties to silk, working them requires different hand skills and loom parts than those used by silk velvet weavers and so those silk velvet weavers we spoke with were reluctant to work on this reconstruction. Instead, the wool and linen handweaver Ruth Gilbert experimented and improvised a velvet technique at her loom, first with plastic straws for rods to create the supplementary pile loops, and later using hollow brass rods. Confirming the generative nature of reconstruction, this strategic challenge prompted new research questions about the invention of mockado: was it made by wool or linen weavers who learned velvet technique, or was it created by silk weavers who experimented with different fibres? Certainly, it required specialist equipment, for when Queen Elizabeth visited Norwich, England’s centre for textile manufacture, she was entertained by a pageant decorated with depictions of seven different looms, one specifically for weaving mockado.^39^

**Fig. 4.**
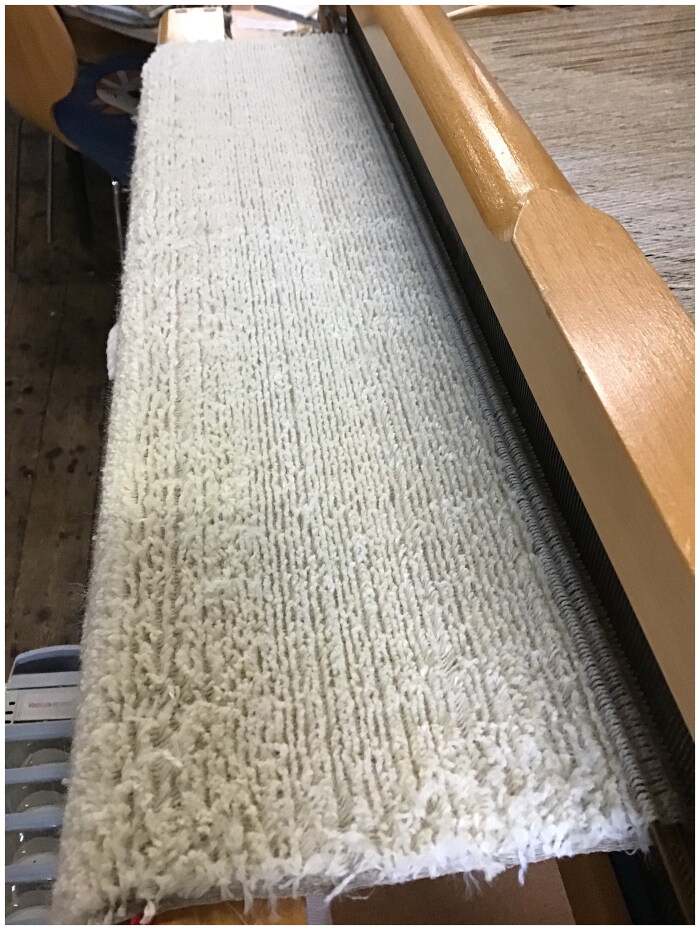
Mockado on the loom, 2021. Photo taken by Ruth Gilbert for the Refashioning the Renaissance Project.

Without inventory information about the lining and structural materials for the doublet, we had to make choices based on other sources. Following examples found in several early seventeenth-century sleeve linings in the SHD collections, we settled on fustian, linen, and hemp interlinings for the doublet, which Gilbert could examine and then weave. Hand-woven linens and naturally-dyed changeable pink and green silk taffeta left over from other SHD projects were repurposed for interlinings and facings. This was done in order to be economical (warping a loom and weaving even small amounts of fabric is costly in both time and money) and following likely Renaissance workshop practice of using what was to hand. Green and pink linings, while not as common as black or red, are found in the Refashioning inventory dataset, and taffeta was a popular choice (appearing in 328 items). Most fabrics required some level of post-loom treatment, which was also done by hand: the fustian was napped with a hand carder, and the hemp stabilised with a gum arabic size. Such processes remind us that cloth taken from the loom was rarely ready to be used straight away by a tailor or seamstress, and that many processes and makers were involved in transforming natural fibres into fashionable textiles (Fig. 4).

## Reconstructing Renaissance Black Dye

**Fig. 5.**
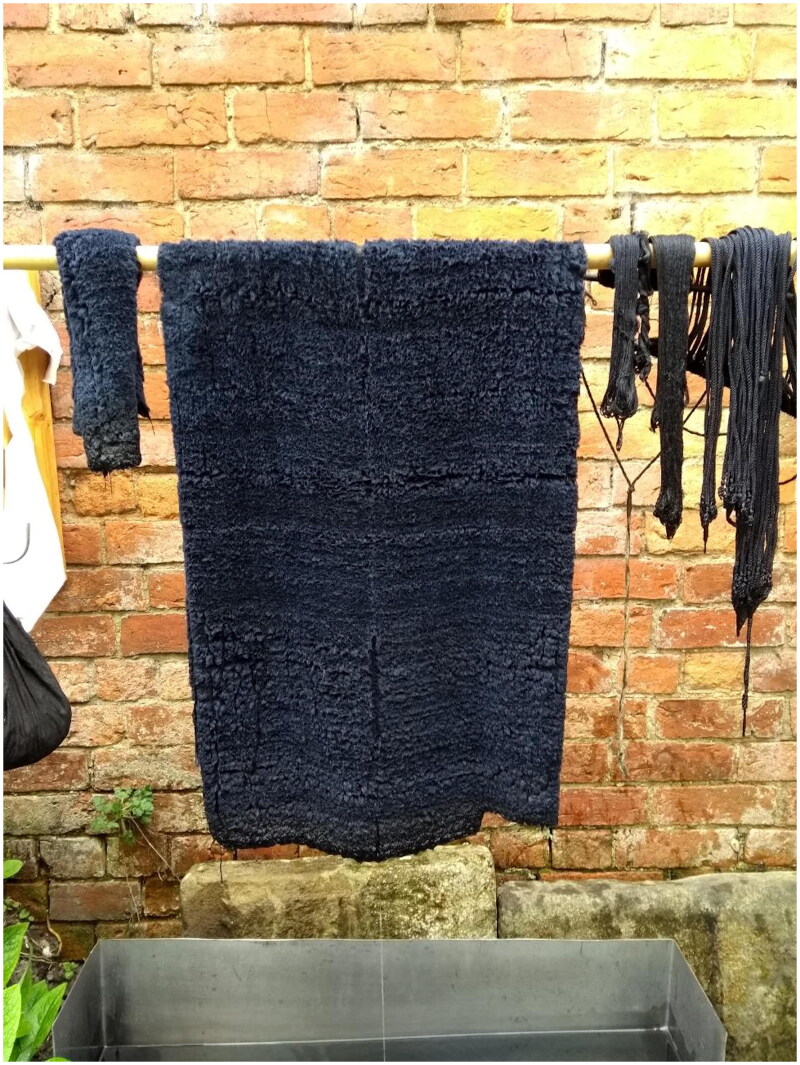
Mockado, braids and silk thread drying after being dyed, 2021. Photo taken by Karl Robinson for the Refashioning the Renaissance Project.

We cannot know which dyestuffs were used for Ristori’s black doublet, so we began with the results of the dye analysis of the SHD wool velvet as inspiration. UHPLC-PDA-HRMS analysis suggested that the SHD wool velvet was likely dyed in one piece.^40^ While most velvets were made with silk that had been dyed in the yarn before weaving, archival sources confirm that mockado was often piece-dyed black.^41^ As it consists of both vegetable and protein fibres, mockado needed to be dyed in multiple steps, to ensure colour adherence to different materials. Woad or indigo was used in the first dyebath, and then the blue cloth was probably treated with an alum mordant before being dipped in a kermes bath. This would likely have dyed the wool successfully, but not the hemp or linen ground. Then the fabric was dyed with black alder and oak gallnuts, a tannin and iron process that would turn the vegetable fibre dark black. A final dip in a logwood and potash dyebath probably gave the fabric a final deeper black hue. This complex recipe, combining more traditional expensive (blue and red dyes), cheap (iron and tannins) and state-of-the-art (logwood) methods shows that early modern dyers used all the techniques available to achieve a good black.^42^ A recipe from Gioanventura Rosetti’s *Plictho* (1548) suggests mixing vitriol with alder bark with iron scale and layering it as ‘when one prepares lasagne’ to create a mixture that can be combined with gall to create ‘excellent’ black dye for both linens and wools.^43^ Due to limits in budget and material availability, we could not dye with costly kermes (it would have cost thousands of pounds to acquire enough kermes to dye our mockado piece) and were unable to source suitable bark from alder trees, so the dyer Karl Robinson chose an alternative method for creating black used by early modern dyers — combining blue and red dyestuffs. Robinson used indigo and cochineal, potent dyestuffs novel to European dyers in the era, rendering a beautiful deep blue-black tone, rather than a true deep black.^44^ Compromises and failures are common in reconstruction projects, and challenges in material sourcing such as this one should be explicitly identified by researchers to explain where concessions were made. Robinson also dyed loop-manipulated braid, made from six looped como silk threads by Beth Trapnell, which decorated the seams and edges of the doublet (Fig. 5).

Hard to dye, often expensive to purchase, and a wonderful contrast to the vibrant colours available on the market, black was a fashionable hue in Renaissance Europe. Scholars have interrogated the sartorial shift towards black in the era and charted its broad symbolic and cultural associations spanning grief, religious strictures, gravitas, but also dignity and status.^45^ While high quality, deep, intense, colourfast blacks were associated with the elites, black was embraced across the social spectrum. Most of the 1143 doublets in the Refashioning database were not described by colour (748 were unlisted and 7 just described as ‘coloured’ or ‘multicoloured’), but of those given a hue, black was by far the most popular (201 items) (other colours, in order of popularity: white, 81; red, 33; brown, 20; purple, 19; grey, 13; yellow, 10; green, 7; blue, 4). Even blacks dyed with lesser materials and techniques that tended to grey, purple, brown, or blue tones could be highly regarded. As the courtier Baldassare Castiglione put it, ‘I am of the opinion that a black colour has more grace in garments than any other; or if not truly black it should at least be of a dark hue’.^46^ In Florence, black was strongly associated with professional classes and merchants, as a colour of loyalty and reliability.^47^ By 1638, this chromatic association with probity was encoded in a Florentine law that required married women of citizen status to cover their colourful skirts, sleeves and bodices with black overgarments after six years, and after twelve years of marriage they were required to dress ‘all in black’.^48^ The waterseller’s choice of black for his doublet (even if his dyer did not achieve a ‘true’ black as we failed to do in our reconstruction) was at once fashionable and respectable, upholding his status as an honest Florentine but also enabling him to set off garments of other colours and participate in urban dress culture.

## Tailoring an Artisan’s Doublet

Robert Dallington, an English visitor to the Medici Court, noted that Florentine fashion was ‘both civil, because black, and comely because fitted to the body’.^49^ Ristori’s inventory does not mention the fit of his doublet, but given his use of fashionable textiles and colour suitable for his status as an artisan, we assumed that the cut would be similarly well chosen. While doublets owned by the elite Medici family who ruled Florence do survive, those from the artisan classes in that city — or indeed any other urban environment across Europe — are largely lost from the material record. A rare example of a doublet associated with a working artisan survives thanks to its later use by the Dutch humanist Hugo de Groot as a disguise when he fled the Netherlands in 1621 (Fig. 6a).^50^ Made of leather, this mason’s doublet was probably more workaday than Ristori’s mockado doublet, and it represents Netherlandish rather than Italian regional style. However, many of its features, such as a collar that folds down and falls open at the neck, reflect doublets seen in depictions of Italian watersellers (see, for example, Fig. 6b), and so this rare survival informed the cut and construction methods used in our doublet.

**Fig. 6a.**
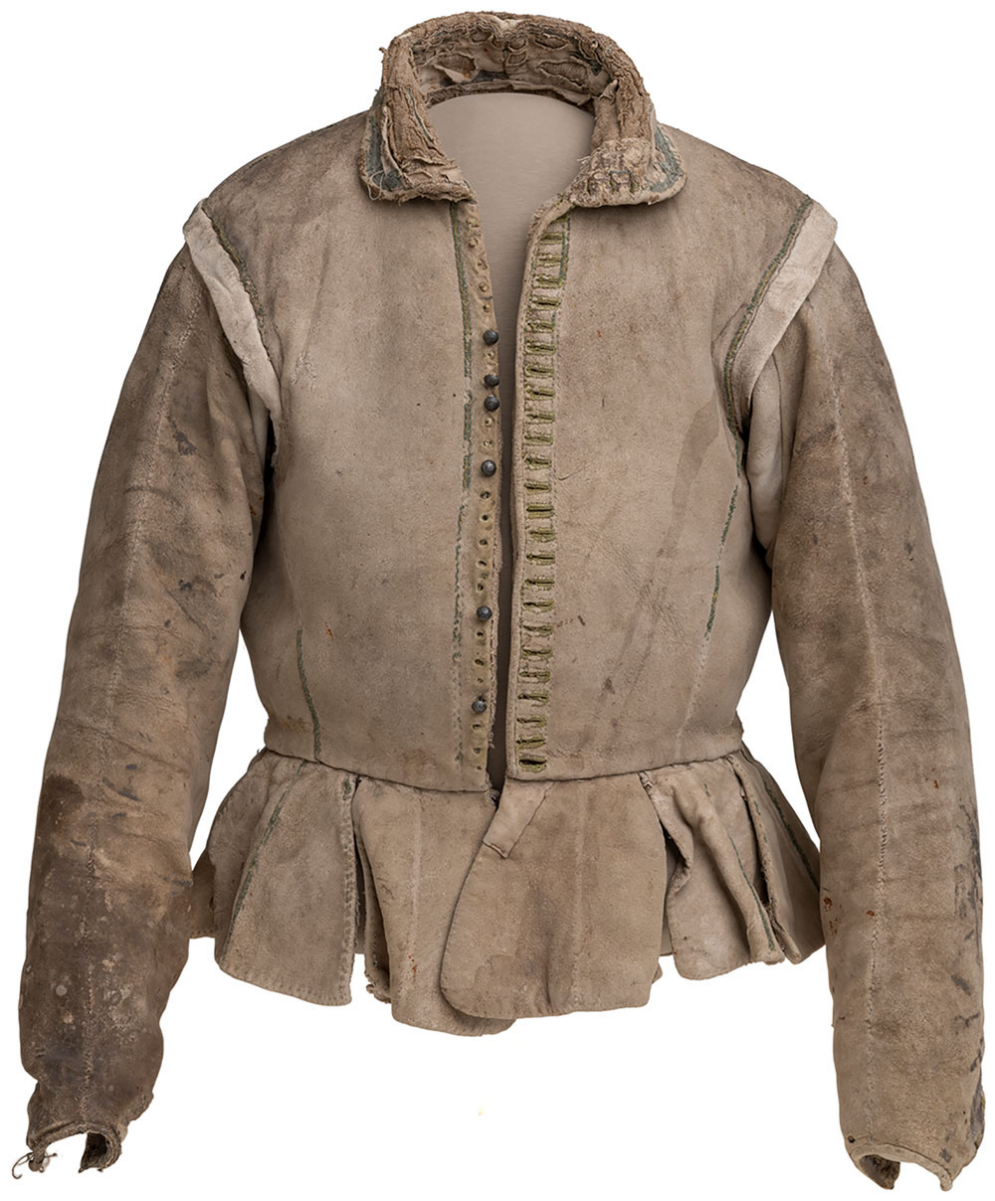
Doublet worn by Hugo de Groot (1582–1645), sheep leather, linen, pewter, 1575–1621, Museum Rotterdam 20535-1. Image in the public domain.

Once again, the scant facts about Ristori’s life posed an interpretative conundrum. While he died in 1631, we were left to wonder whether his mockado doublet was a recent purchase reflecting contemporary fashion, or if it was in ‘nasty’ condition and kept in a trunk because it was older and outmoded? We decided to create a sleeveless doublet, in part to economise on the mockado, but also because Ristori’s inventory mentioned eight pairs of detachable sleeves, and did specify that one white leather doublet was found ‘with its sleeves’. Sleeveless doublets were often made with eyelets around the armhole through which laces could attach sleeves, enabling easy movement and flexible dressing to suit shifts in both weather and fashion.

Using her experience as a trained tailor, and her first-hand observations of many early modern doublets, Melanie Braun cast pattern shapes using a compass and proportional measures. Using geometry, the shape of the reconstructed doublet accounted for the physical body requirements of the model, historian Valerio Zanetti, while giving him an elegant and idealised form.^51^ After dyeing, Claire Thornton carefully laid out Braun’s paper pattern pieces on the woven mockado, leaving barely any offcuts and even using piecings in order to create the pattern (both an early modern practice of economy and a contemporary necessity when we had limited funds to commission mockado) (Fig. 7).

**Fig. 6b.**
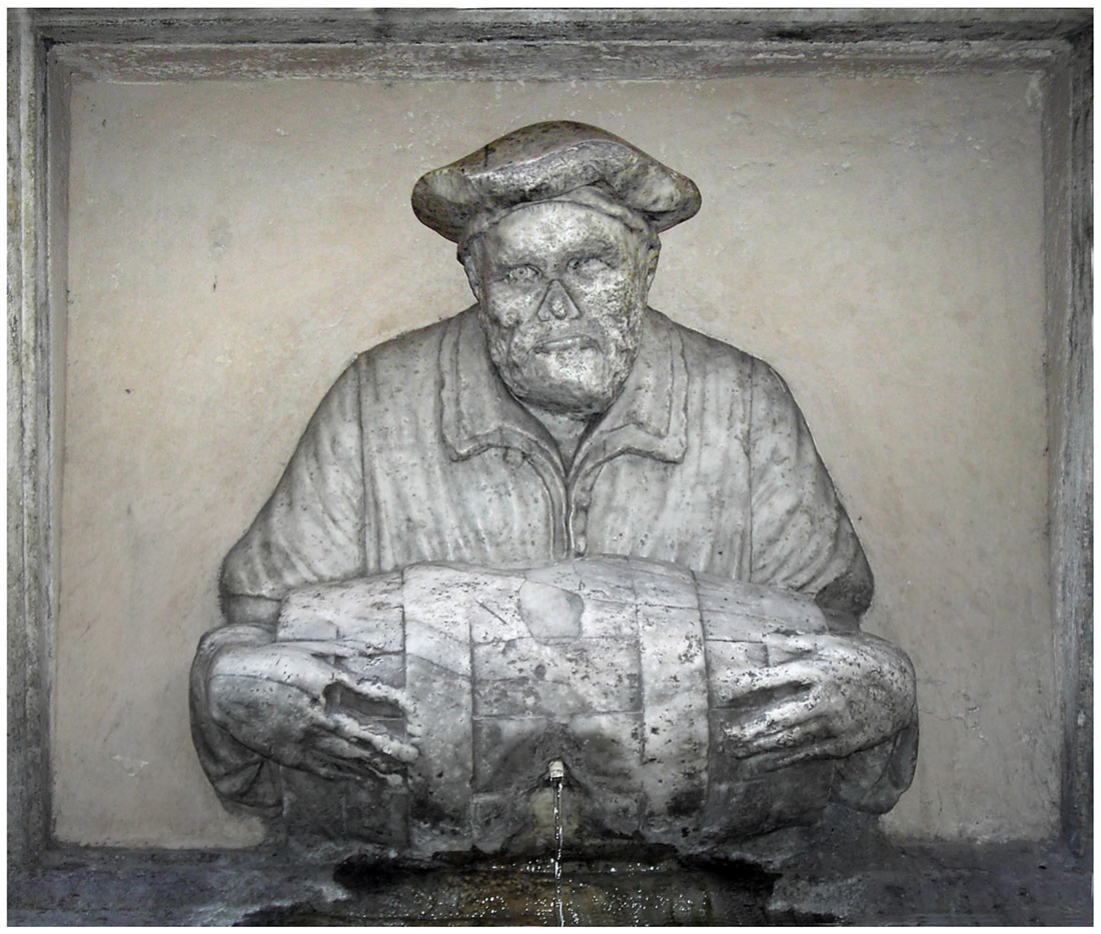
Jacopo del Conte, *Il Facchino*, originally located on Via del Corso, now on Via Lata (since 1872), *c.* 1580. Image in the public domain.

The fabric and dyestuffs continued to present challenges throughout the construction process. Underneath the mockado, the doublet is a complex construction of linings and padding made of hemp, wool (woven and roving), linen, fustian, and synthetic whalebone (Fig. 8). Much structure also comes from pad stitching and hand manipulation as the tailor works. Jordan Colls, who assembled the doublet, explained that the ‘trickiest and most time-consuming’ element of work was responding to the hand-woven fabrics, particularly the mockado, which had a very tight weave in certain areas.^52^ It required significant amounts of manipulation and finishing as the doublet was assembled; seam allowances were shaved down flat and pressed with a hot iron, and the fabric had to be combed and shaved to remove felting that had occurred during the dye process to ensure an even appearance. Not only was this slow work, but it was also messy: the dye kept transferring onto hands, and a lot of wool dust was created, reminding us of the often dirty and dangerous working conditions endured by early modern textile workers. During fittings, the mockado was so fluffy and saturated with excess dye that both pile and dye transferred onto the handmade white linen shirt. This issue was solved by the application of silk inkle lace to bind the armhole edges.

**Fig. 7.**
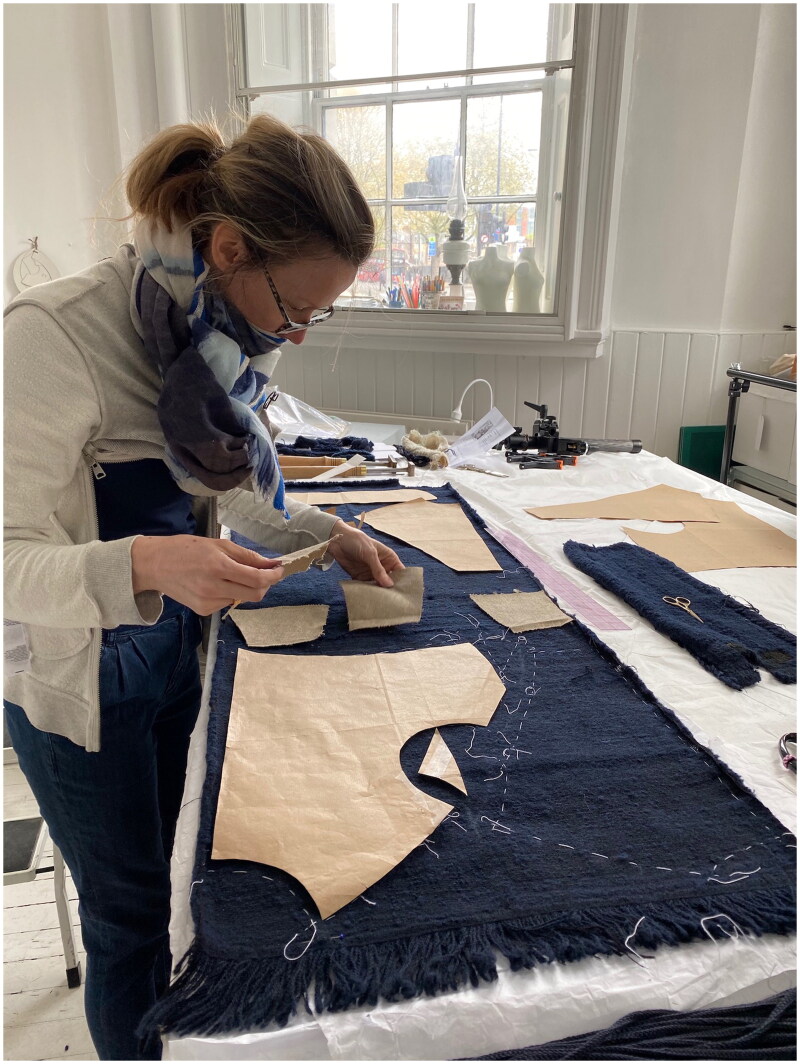
Claire Thornton laying out the pattern on the mockado, 2021. Photo taken by the School of Historical Dress for the Refashioning the Renaissance Project.

## Stamping the Doublet

Ristori’s doublet is described as ‘stamped’, which suggests that its decorative pattern was not created during the weaving stage but rather was applied after the fabric had been taken from the loom and out of the dye vat. While complex woven patterns are time-consuming and costly to produce, stamping is comparatively quick and easy to execute, even by an unskilled practitioner. Pattern is created through the application of heated metal tools that emboss motifs into the surface of the textile. This technique was often executed by hand, but was also mechanised on a rolling press, and an example for printing and embossing stuffs like velvet vests is depicted in Denis Diderot and Jean le Rond d’Alembert’s *Encyclopédie ou Dictionnaire raisonné des sciences, des arts et des métiers* (1751–1780).^53^ While it is easy to scorch the fabric or by contrast fail to make a clear impression due to a lack of sufficient heat or pressure, even as first-time experimenters we were able to quickly create successful patterns in offcuts of silk velvet by heating forks over hotplates and then impressing the tines into the pile. Stamped textiles survive in many museum collections, suggesting that the technique was widely used, particularly on mixed-fibre velvets like mockado (Fig. 9). Of the fifteen stamped items (including sleeves, petticoats, edgings, doublets, and a gown) in the Refashioning database, eight were mixed fabrics like *ciambellotto* and mockado, while three were silk velvet.

**Fig. 8.**
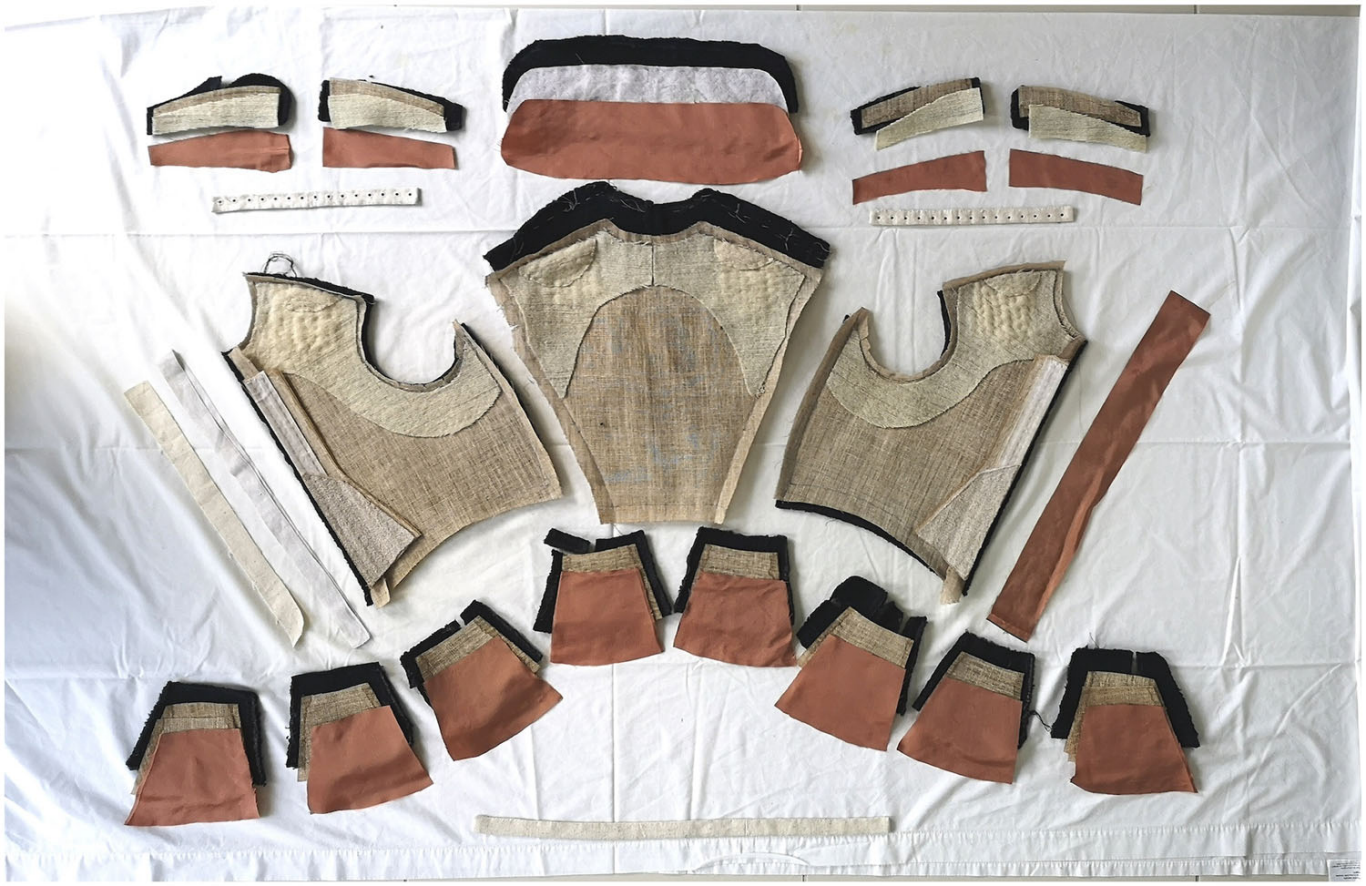
The cut pieces of the doublet laid out prior to assembly, 2021. Photo taken by the School of Historical Dress for the Refashioning the Renaissance Project.

In the absence of any detail about the stamped pattern on Ristori’s doublet, we followed the motifs found on a surviving stamped crimson velvet in the SHD collections. Metalworker Dave Budd cast two stamps, one with a double ‘S’ and six-ball flower motif, and the other a four-petalled flower. The creation and control of heat is often a challenge for historical reconstruction, and in this case Colls and Tiramani proceeded with caution to avoid burning the mockado, using an electric hotplate to heat the tools (Fig. 10). Unfortunately, the pile of the mockado was several millimetres higher than the design of the double S stamp, and so it simply impressed a rectangle rather than a legible design into the fabric. Nevertheless, the flower stamp was successful, and working by eye (as many surviving examples seem to suggest), a striking square and bar strapwork pattern transformed the mockado into a more three-dimensional fabric that catches the light.

**Fig. 9.**
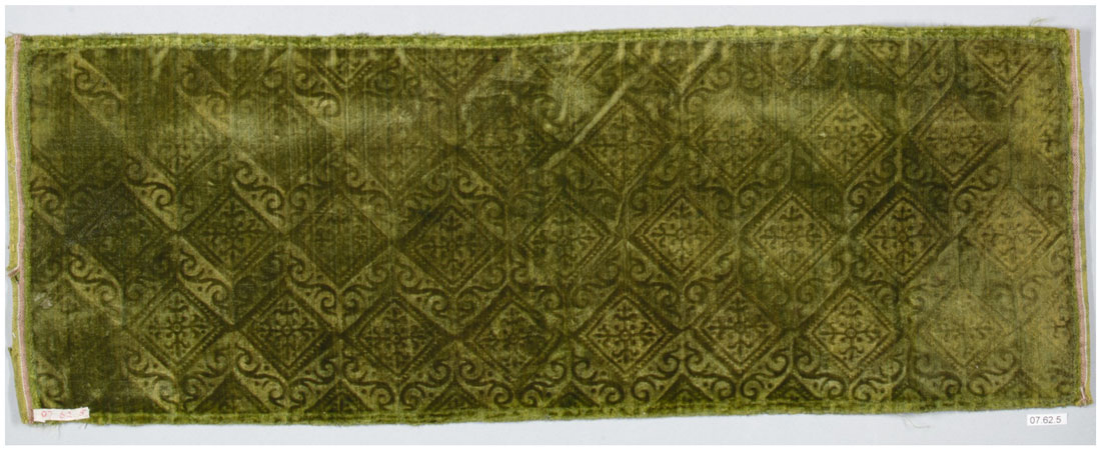
Stamped wool velvet, sixteenth–seventeenth century, Italian?, Metropolitan Museum of Art, 07.62.5. Image in the public domain.

## Bringing the Doublet to Life

While the doublet was the focus of the reconstruction, Ristori’s doublet was one element of an outfit and could not have been worn alone. From his inventory, we can build a picture of the entire wardrobe owned by a middling artisan and see the doublet in relation to his overgarments, lower-leg garments, and undergarments. The doublet, therefore, was made in ways that accommodate other items of clothing. Handmade metal hooks and eyes were sewn in to attach the doublet to hose, and eyelets were used for lacing bands at the shoulders so that sleeves could be attached to the doublet (Fig. 11). A pair of hose, sleeves, and a shirt were made for this reconstruction, in order that the doublet could be worn attached to and over the closest garments worn on Ristori’s body, and a full outfit (comprising stockings, shoes, a swordbelt, and hat) was borrowed from SHD for dressing and photography. Only when worn over a shirt and attached to sleeves and hose do we get a full sense of the range of motion, effect of movement and overall look of the waterseller’s doublet. Our model, Valerio Zanetti, was selected to represent Ristori not only for his Italian heritage, but also because as a scholar of embodiment and clothing he could reflect on the experience of being measured, fitted, and dressed in tailor-made early modern fashions (as is explored in another article in this issue) (Figs. 12a and b).^54^

**Fig. 10.**
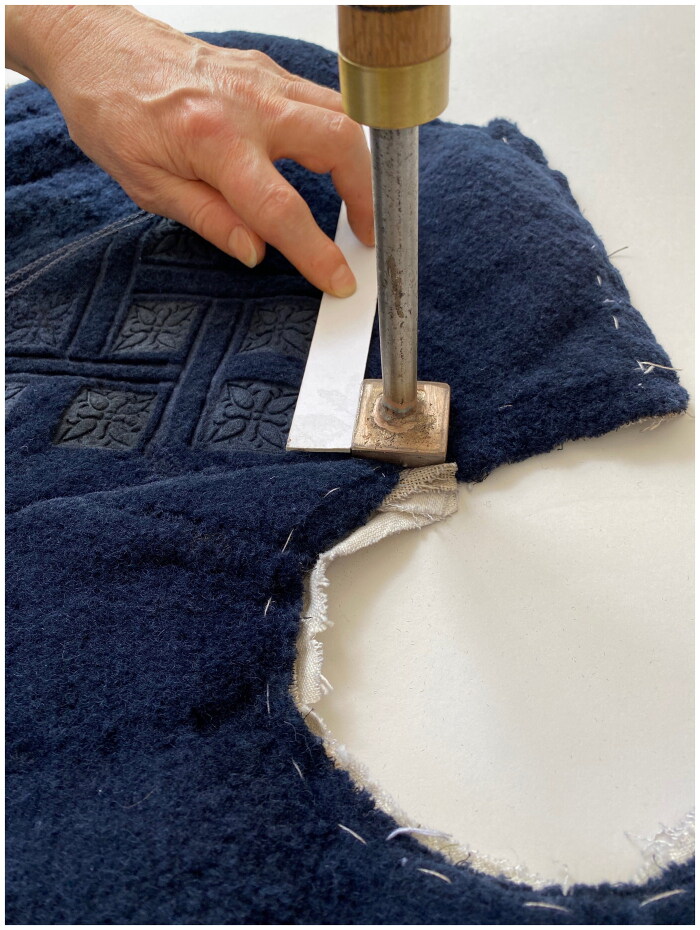
Stamping mockado using a heated four-petalled flower stamp by Jordan Colls and Jenny Tiramani, 2021. Photo taken by the School of Historical Dress for the Refashioning the Renaissance Project.

**Fig. 11.**
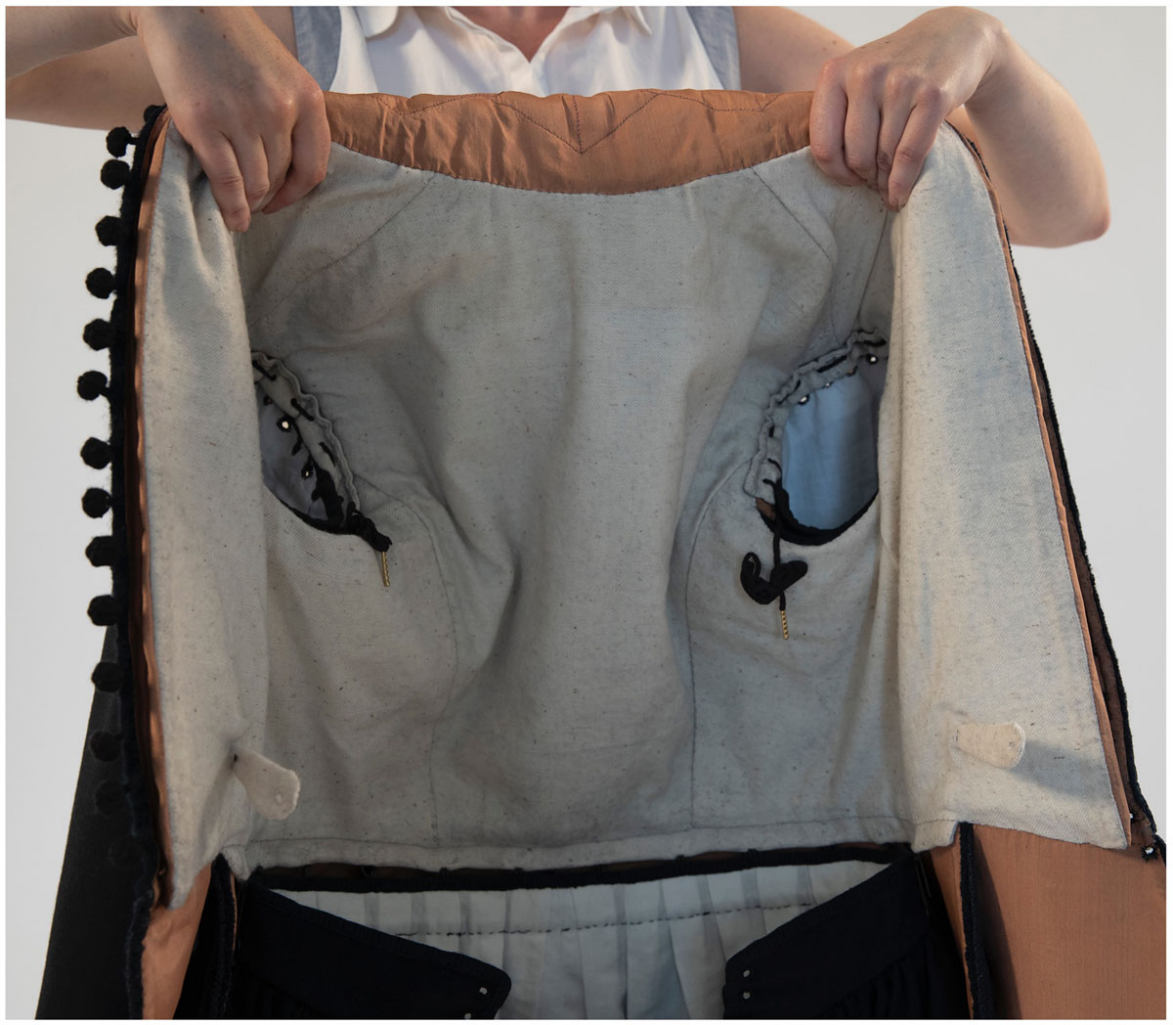
The inside of the doublet, which is pointed (attached) to the hose. Note the napped fustian lining and the eyelets at the armholes to attach the sleeves. Photo taken by Ana de Matos for the Refashioning the Renaissance Project.

**Figs. 12a (left) and b (right).**
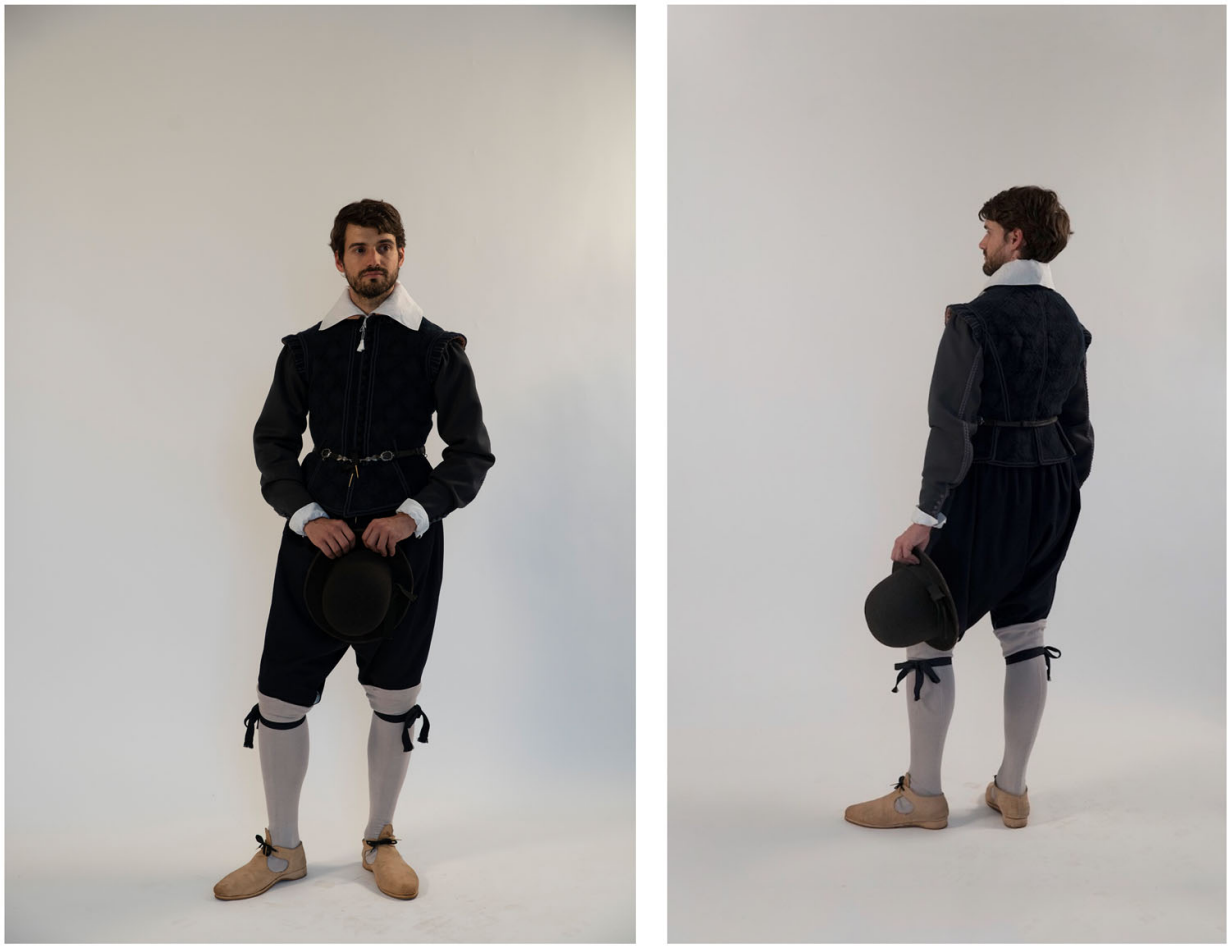
The reconstructed waterseller’s black mockado doublet, front and back. Modelled by Valerio Zanetti. Photo taken by Ana de Matos for the Refashioning the Renaissance Project.

Each time the doublet is worn, we observe its stiff structure soften with the heat of the body, and the stamped decoration has already started to grow fainter as the springy wool fibres relax and wear down in areas where the mockado is rubbed, such as at the sides under the arms. The doublet, in its material form, is an active object that has life in motion and will age, fade, and wear with use (although we will store it in archive boxes and acid-free tissue paper when it is not being worn or on display).

Reconstructions are best done as iterative processes — and many of the makers expressed a deep wish to have a second chance — equipped now with a better idea of how to improve on their first attempts and inspired to develop their technique. With time and funding to repeat this experiment, particularly now pandemic restrictions have eased, we would be able to nuance and improve note-taking and observations of the process, revisit the archives with more questions, and create a more evenly woven mockado in deeper black dye with a more defined stamped pattern. Certainly, this is a challenge for academic reconstruction projects, which require unusually high funding for the humanities and modes of collaboration that are often unsupported outside of the sciences; funding bodies prefer novelty in project proposals, and makers and researchers alike are sometimes reluctant to admit compromises or failures in their work. While our reconstructed doublet was likely made with far more time, care, critical attention, and expense than Ristori’s doublet, we must remember that his would not have been an experimental attempt.

What we have created is a historical possibility that to the best of our knowledge and abilities reconstructs a version of Ristori’s black stamped mockado doublet. The finished doublet has been used for exhibition display, teaching, and public talks, offering a haptic experience for researchers who can engage with the object up close.^55^ While material reconstructions often conceal processes of making, digital methods of display such as a video of the dressing process and a 3D animation of the construction offer alternative ways of reconstructing the waterseller’s doublet.^56^ The process of reconstruction was just as important as the finished object in generating new questions of archival, textual, visual, and material sources and encouraging close-looking and imaginative scholarship. Each of the words describing Ristori’s doublet prompted new research into early modern clothing. The findings of this research are materially manifested in the doublet, embedded in the making practices of all those who worked on the garment, and published in scholarly outputs such as this article.

## Conclusion

This experiment asked what happens when we try to reconstruct an object that we know existed but that does not remain in the visual or material record. As researchers we are often led by chance survival. Our work can be prompted by a particularly compelling source that frames our research questions — it may be an intriguing text, detailed image or captivating object. The doublet reconstruction project offers a creative and generative approach to the widespread challenge faced by researchers whose sources are missing, limited or problematic.

In many ways, Ristori’s doublet is as close to a seventeenth-century fashionable artisan’s doublet as we can hope to get in the twenty-first century. From fibre to finished garment, the doublet was made entirely by hand by skilled makers using historically appropriate materials. But there are limits to its historicity: we had to compromise when raw materials were unavailable or prohibitively expensive, it was made in modern conditions with electric lights and heating by people who were attempting some of these techniques for the very first time.^57^ It also cost far more than Ristori would have spent on a garment, and much of our cost went not on raw materials (which would have accounted for most of the price of any item of clothing in the seventeenth century) but on makers. There is much we can never know about Ristori’s doublet.

With only seven words written by an inventory appraiser to lead the experiment, the finished object should be considered an imaginative reconstruction informed by rigorous archival, visual, material, and scientific research. Silences in the archives prompted creativity, spurred on new research questions and encouraged close reading of sources that were available to us. We had to approach scant inventory information creatively. From the very start, questions immediately arose that took us in all directions of economic, social, legal, and cultural history: how old might Ristori have been when he died, and when might the doublet have been made? He died in a pandemic year, and left behind young children, so he was likely no older than middle-aged. But was his doublet in a ‘nasty’ condition because it was very old, or second-hand, and so should we make it in a style from a few decades before the date of the inventory, or was it something that was more recently made but he had worn to work and was damaged through repeated daily use and splashed by water? Why would a waterseller want to look fashionable? What would he have been legally allowed to wear? What was mockado exactly, and how was it made? Such questions led us back to archives and libraries to research Ristori’s neighbourhoods and the labour and social standing of watersellers, and to scrutinise other objects in his inventory for more clues to the habits and mentalities of a Florentine waterseller with a small but carefully chosen wardrobe.

Even though the record is sparse, it is provocative. Ristori was certainly not a rich man — he does not seem to have been a citizen or head of household, but he did participate in the middling version of late Renaissance Florentine culture. Artisans could have significant sums of money invested in household possessions, even while holding a comparatively low economic and social status.^58^ Ristori owned forks as well as knives (a relative novelty in the culinary material culture of the period) and decorated his home with paintings of beautiful Florentine women as well as Jesus and the Saints. He displayed terracotta busts and even four angels made of *cartapesta* (papier-mâché). Perhaps Ristori admired his appearance in the two convex mirrors that he kept in a box in his bedroom alongside a sculpture or doll representing the baby Jesus and a receipt for seventeen spoons and forks pawned by his wife. What might his black stamped mockado doublet have meant to him? Did he wear it with his green pair of sleeves, or did he mix and match with the other seven pairs stored in his house? Did he prefer it to his other four doublets — made of white leather, Nimes wool, black spun silk, and black baize?

**Fig. 13.**
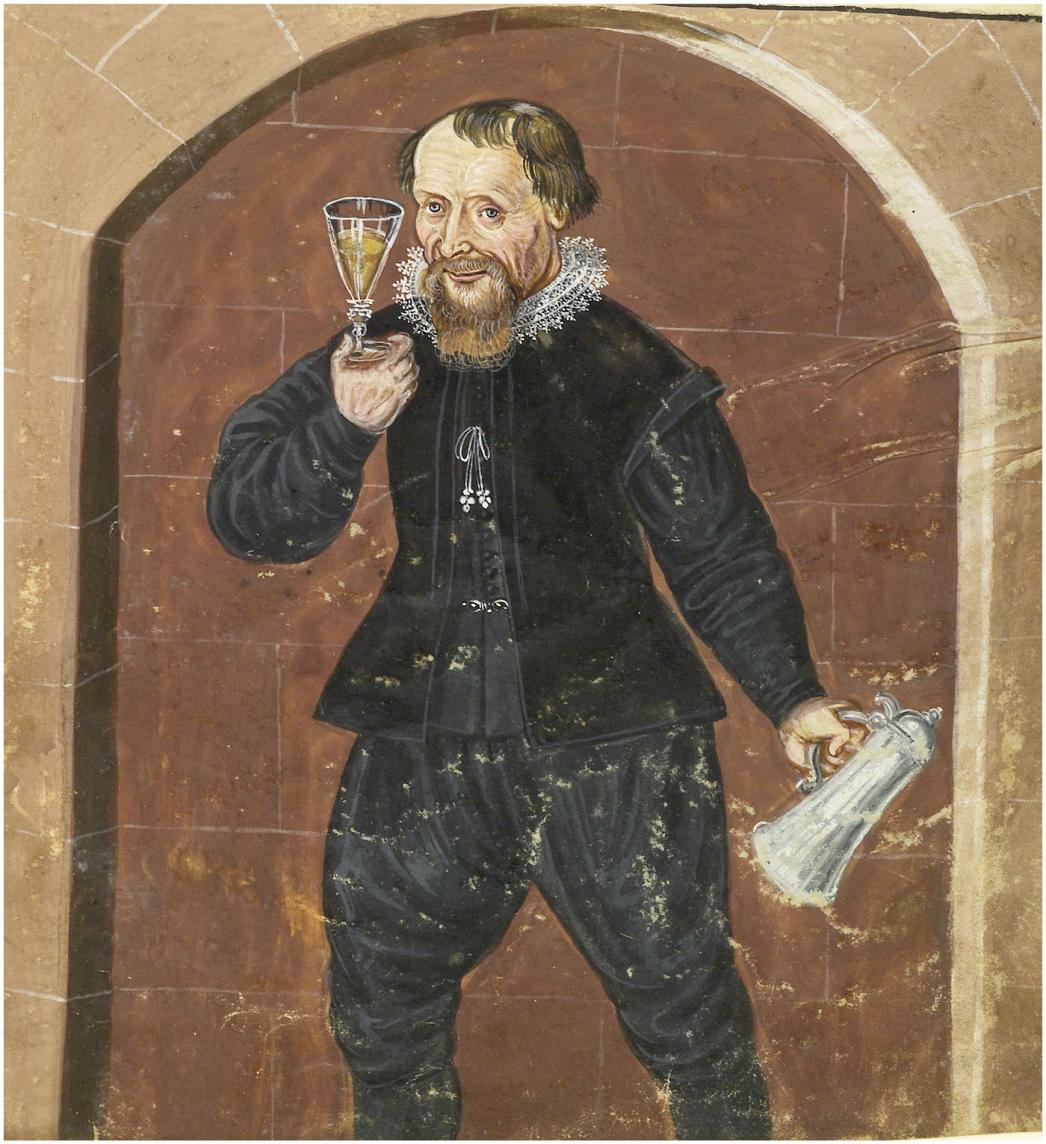
Anonymous, Hanns Lebender the Waiter from *The Housebooks of the Nuremberg Twelve Brothers Foundation*, 1641. Stadtbibliothek Nürnberg. Image in the public domain.

By giving a material presence to the waterseller’s doublet, we call attention to the fashionable aims and innovative techniques used by the artisan classes to participate in Renaissance clothing culture. While more work needs to be done to explore distinctive sartorial groups and subcultures among artisans, this doublet represents a scholarly and physical intervention that shows that early modern artisans could express themselves fashionably through textiles and clothing. We restore Ristori and his fellow artisans’ reputations as discerning dressers living, working, and shopping in a dynamic urban environment in which novel and carefully chosen textiles, creative and skilled makers, and a culture of materially literate consumers combined to generate a lively middling material culture of Renaissance fashion. Ristori’s doublet, an extraordinary object that represents many state-of-the art aspects of seventeenth-century everyday fashion, becomes a lens through which to explore the craft and culture of early modern Europe (Fig. 13).

**Fig. 14.**
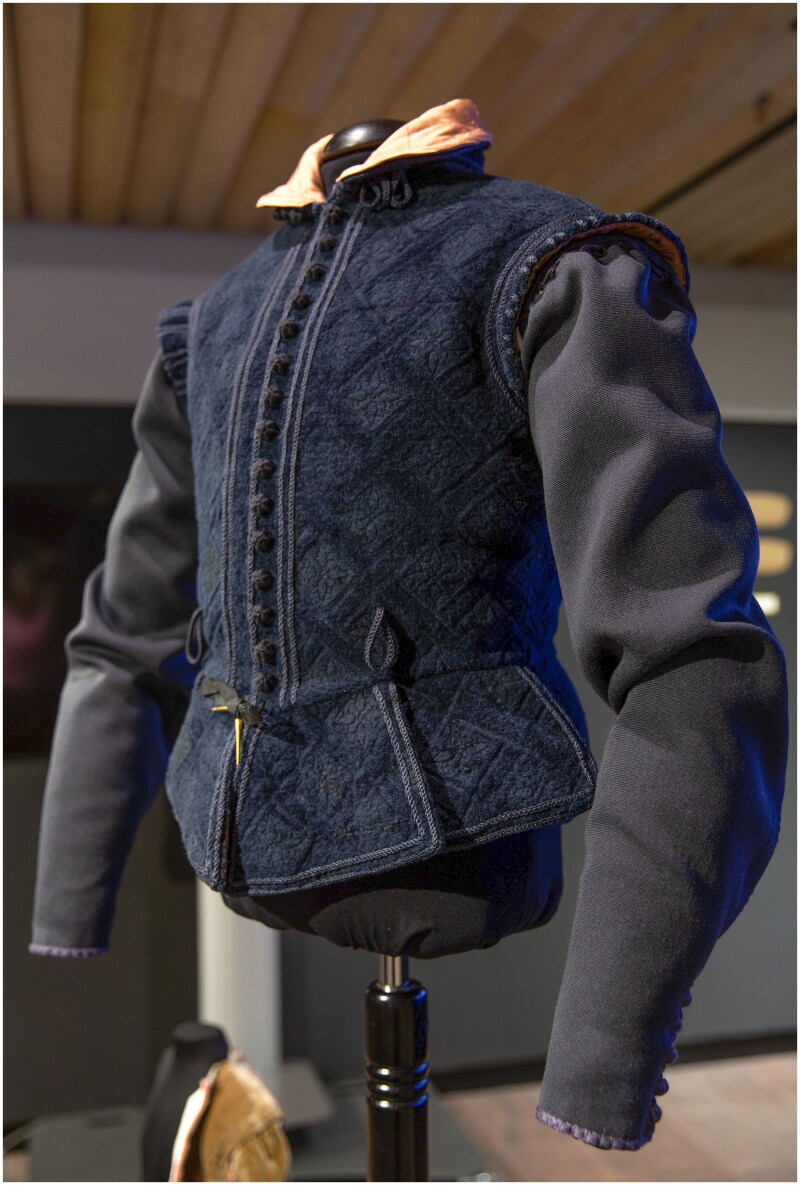
The reconstructed waterseller’s doublet, mounted for exhibition at Aalto University, 2021. Photo taken by the Refashioning the Renaissance Project.

Francesco Ristori is just one of 448 artisans who left a little historical trail that was picked up by the Refashioning Project. We could have selected any of the other artisans and reconstructed any of the over 80,000 items in the database, each of which would likely have led down divergent yet similarly fascinating paths of research, raising many new questions and posing different and likely stimulating challenges too. Reconstructions can help us ask new research questions and they are also something of a political intervention. They foster collaboration and disrupt hierarchies of knowledge. They enable us to better appreciate skills of makers past and present and encourage us to revisit, revalue, and revive lost or vanishing crafts. Reconstructions can help us to reframe the scholarly focus and enable us to consider underexamined subjects and create new pedagogical tools to offer sensory experience and communicate our research in experimental formats such as academic articles that outline process in the first person, as well as in videos and digital animations.^59^ By giving what has been lost to the ravages of time and source bias a material presence, we make the overlooked visible (Fig. 14).

